# Risk of Pleural Recurrence in Early Stage Lung Cancer Patients after Percutaneous Transthoracic Needle Biopsy: A Meta-analysis

**DOI:** 10.1038/srep42762

**Published:** 2017-02-16

**Authors:** Ting Wang, Lili Luo, Qinghua Zhou

**Affiliations:** 1 Lung Cancer Center, West China Hospital, Sichuan University, Chengdu, Sichuan, China; 2 Department of Medical Oncology, Sichuan Cancer Hospital & Institute, Sichuan Cancer Center, School of Medicine, University of Electronic Science and Technology of China, Chengdu, China; 3 Department of Pediatrics, West China Second University Hospital, Sichuan University, Chengdu, Sichuan, China

## Abstract

Percutaneous transthoracic needle biopsies (PTNB) are widely used for the diagnosis of a peripheral pulmonary nodules, but the risk of pleural recurrence in lung cancer patients remains undetermined. Our meta-analysis aims to answer the question whether PTNB strategy increases the risk of recurrence. PubMed, EMBASE, The Cochrane Library, and China National Knowledge Infrastructure (CNKI) were searched from inception to January 9, 2016. A total of 1242 patients from 5 studies were included. The results showed that PTNB does not increase risk of total recurrence (Odds Ratio,0.78; 95% CI, 0.53 to 1.15) or pleural recurrence (Odds Ratio,1.58; 95% CI, 0.41 to 6.12) compared with non-PTNB strategies in early stage lung cancer patients. Subgroup analysis showed that PTNB was associated with increased pleural recurrence (Odds Ratio, 10.76; 95% CI, 2.92 to 39.70) in patients with sub-pleural lesions but not in patients without sub-pleural lesions (Odds Ratio, 0.96; 95% CI, 0.24 to 3.89). In conclusion, PTNB should not be recommended in lung cancer patients with sub-pleural lesions. However, PTNB is recommendable to use in other patients without sub-pleural lesions to whom pathological diagnosis is necessary, especially in patients with relevant comorbidities.

Percutaneous transthoracic needle biopsies (PTNB) are widely used for the histologic diagnosis of a peripheral indeterminate pulmonary nodule and reported a high diagnostic yield of 90% sensitivity and 97% specificity^1^,^2^. However, pleural recurrence was also reported as a long term complication of PTNB in many literatures^3^,^4^,^5^,^6^. Recent evidence focusing on recurrent outcomes after PTNB was controversial^7^,^8^,^9^,^10^,^11^. In 2005, Matsuguma *et al*. reported that PTNB could cause a higher pleural recurrence rate (9.1%) than bronchoscopy biopsy and sputum (1%) in patients with resected stage I non-small cell lung cancer^7^. In 2011, Inoue M *et al*. also reported that PTNB might increase the risk of pleural implantation in stage I lung cancer patients, especially patients with stage IB disease^8^. However, the pleural recurrence of early stage patients with lung cancer reported in other studies were not affected by PTNB^9^,^10^,^11^.

The evidence on this topic is controversial. Although several reviews of PTNB have been published, most of them focused on the diagnostic yield and incidence of short term complications such as pneumothorax or hemorrhage^1^,^12^,^13^. We therefore conducted this meta-analysis to assess whether PTNB strategy will increase the risk of pleural recurrence.

## Methods

### Eligibility criteria

This meta-analysis was performed according to the PRISMA (Preferred Reporting Items for Systematic Reviews and Meta-Analyses) statement and MOOSE (Meta-analysis Of Observational Studies in Epidemiology) guidelines^14^,^15^. Randomized clinical trials (RCTs) and cohort studies, being published from 1966 to November 9, 2016, which reported comparisons of recurrence or pleural recurrence between patients diagnosed by PTNB or other invasive techniques, such as open surgery, video assisted thoracoscopic surgery or bronchoscopy biopsy etc., were included. The study participants were patients with histologically or cytologically confirmed stage I lung cancer. The main intervention was PTNB of all types, such as CT-guided PTNB or ultrasonography-guided PTNB. The studies focusing on transbronchial needle aspiration or intraoperative needle biopsy would be excluded.

### Search strategy

An electronic search in PubMed, EMBASE, The Cochrane Library, and China National Knowledge Infrastructure (CNKI) were conducted from 1966 to November 9, 2016 by two investigators (Ting Wang and Lili Luo). The following key words in combination as medical subject heading terms and text words were used: “lung cancer”, “percutaneous transthoracic needle biopsy”, “pleural recurrence” OR “recurrence”. Potentially relevant articles were identified by reading titles and abstracts. The full texts of the relevant articles were read to determine whether they met the inclusion criteria. The references were also searched to identify relevant studies. Only studies published in English were searched and included. For studies without outcome data mentioned above, the corresponding author was contacted.

### Quality assessment

For RCTs, methodological quality was assessed using the five point Jadad scale. For cohort studies, the 9-star Newcastle-Ottawa Scale (NOS) was used to assess the risk of bias^16^,^17^. The NOS scale is an 8-item instrument that allows for assessment of patient population and selection, study comparability, follow-up, and outcome. Interpretation of the scale is performed by awarding points for high-quality elements. Studies with 5 or more stars were defined as high-quality studies and were included. Quality assessment was performed by two investigators independently (Ting Wang and Lili Luo). Any disagreement will be present to discuss within all authors.

### Statistical analyses

A unified form for data extraction was used. Study information including author name, study year, sample size, tumor size and event number in each group were collected. The pooled odds ratio was used to compare the recurrence rate or pleural recurrence rate between PTNB group and non-PTNB group. The pooled OR and its 95% confidence interval (CI) were calculated using the Z test, along with 95% CIs. Statistical heterogeneity between studies was examined using the Cochrane Q test by calculating the I^2^ value^18^. An I^2^ value greater than 50% or p value less than 0.05 were considered to represent significant heterogeneity. For RCTs, the pooled HR and the 95% confidence interval (CI) were calculated using the Mantel-Haenszel formula (fixed-effect model) when heterogeneity was not detected (p > 0.05), or using the DerSimonian-Laird formula (random-effect model) when heterogeneity was significant (p < 0.05)^19^. For cohort studies, the DerSimonian-Laird formula (random-effect model) were used. Publication bias was evaluated using the funnel plot and the Begg’s test^20^. Statistical analyses were performed using RevMan5.3 software (the Cochrane Collaboration, Oxford, England).

## Results

### Study selection

**Figure 1:**
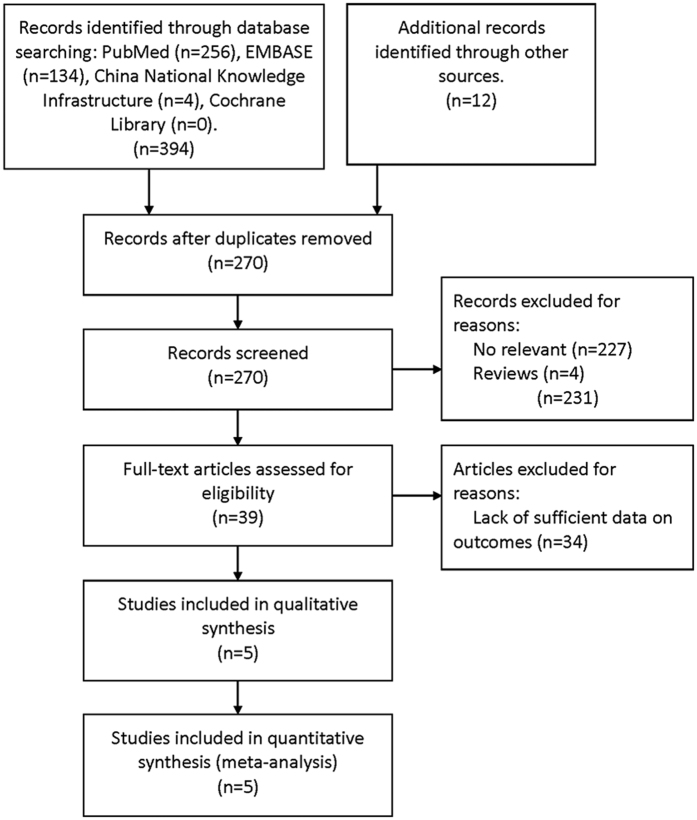
Flowchart of the process for the identification of relevant studies.

Electronic search identified 394 potentially relevant references. Additional 12 references were further identified by checking the reference list. After excluding duplicate and irrelevant references through reading the abstracts, 39 references were read in full and 34 references were excluded for lack of data either on recurrence or pleural recurrence. Finally, five references fulfilled the inclusion criteria and provided data for the meta-analysis^7^,^8^,^9^,^10^,^11^. Figure 1 shows the flowchart of the search results.

### Characteristics of included Studies

According to the searching result, no RCTs fulfilled the inclusion criteria. All five included articles were cohort studies published from 2005 to 2016^7^,^8^,^9^,^10^,^11^. This study included 1242 patients and contained four studies from Asia (Japan)^7^,^8^,^9^,^11^ and one study from Europe (Germany)^10^. Potential confounders, such as tumor stage, tumor size, age, gender, history of smoking, histological type and surgical approach were reported and adjusted in some of included studies. The quality score of included studies ranged from 6 to 8 stars. Characteristics of the included studies are listed in Table 1.

### Impact of PTNB on total recurrence

**Figure 2:**
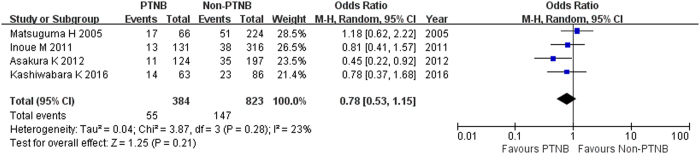
Forest plot showing the impact of PTNB on total recurrence. *CI: confidence interval, PTNB: percutaneous transthoracic needle biopsy.

Four studies reported the recurrence between PTNB and non-PTNB groups^7^,^8^,^9^,^11^. Significant heterogeneity was not found among studies (I^2^ = 23%, p = 0.28). Random-effect model was used to perform the meta-analysis of cohort studies. The pooled OR estimate was 0.78 (95% CI, 0.53 to 1.15; p = 0.21; Fig. 2), which means PTNB is not associated with recurrence in stage I lung cancer patients.

### Impact of PTNB on pleural recurrence

**Figure 3:**
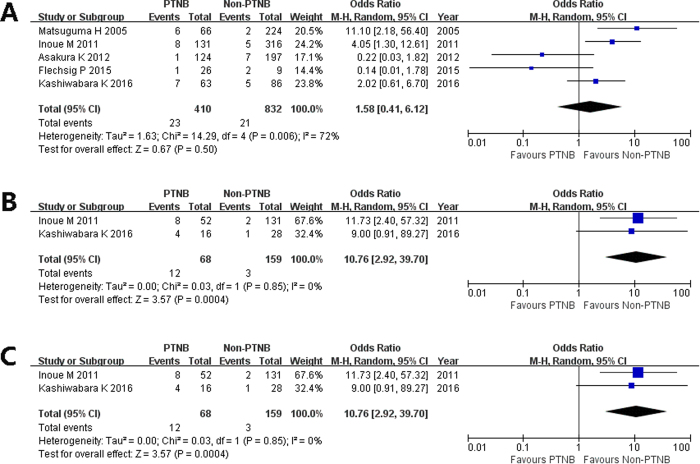
Forest plot showing the impact of PTNB on pleural recurrence and forest plot of subgroup analysis according to tumor location. *CI: confidence interval, PTNB: percutaneous transthoracic needle biopsy.

Five studies reported the pleural recurrence between PTNB and non-PTNB groups^7^,^8^,^9^,^10^,^11^. Significant heterogeneity was found among studies (I^2^ = 72%, p = 0.006). Random-effect model was used. The pooled OR estimate was 1.58 (95% CI, 0.41 to 6.12; p = 0.50; Fig. 3A), which means PTNB is not associated with pleural recurrence in stage I lung cancer patients. Subgroup analysis according to the tumor location was performed. In patients with sub-pleural lesions, the pooled OR estimate was 10.76 (95% CI, 2.92 to 39.70; p = 0.0004; Fig. 3B). In patients without sub-pleural lesions, the pooled OR estimate was 0.96 (95% CI, 0.24 to 3.89; p = 0.96; Fig. 3C). These results showed that, for early stage lung cancer, PTNB will increase the risk of pleural recurrence in patients with sub-pleural lesions but not in those without sub-pleural lesions.

### Publication bias

**Figure 4:**
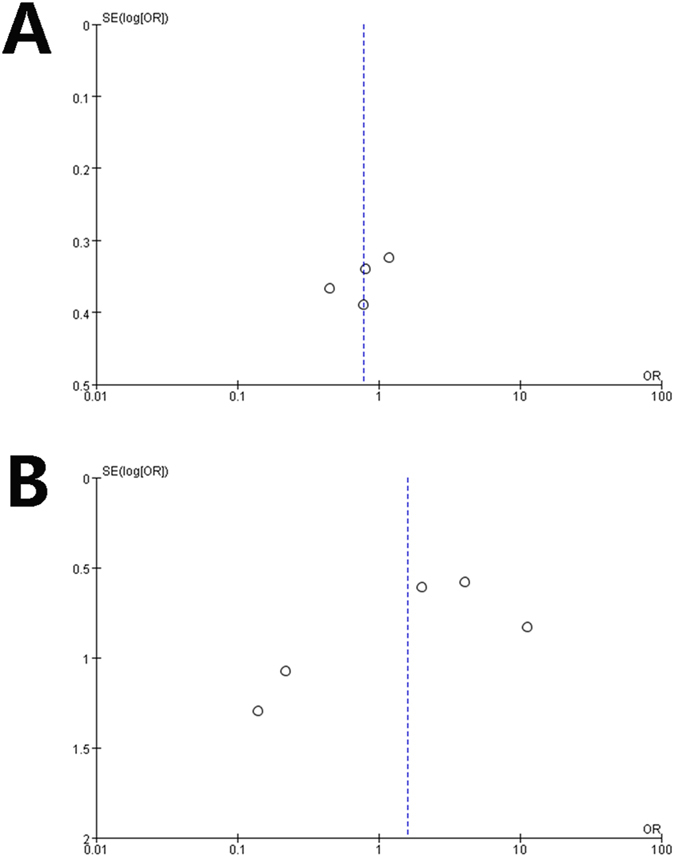
Funnel plots showing the publication bias of total recurrence and pleural recurrence.

Visual inspection of the funnel plots did not show the asymmetry typically associated with publication bias. Evidence of publication bias was also not seen with the Begg’s tests of total recurrence (p = 0.309, Fig. 4A) and pleural recurrence (p = 0.462, Fig. 4B).

## Discussion

For pulmonary nodules, histopathological diagnoses are often important before appropriate therapeutic strategies are performed. For the recent decade, PTNB has been developed one of the most common diagnostic tools in the management of such nodules, especially peripheral lung cancers^1^,^2^. Despite high diagnostic accuracy and low short term complications have been widely studied for years, it is necessary to understand the long term comorbidities like pleural recurrence related to diagnostic procedure. Some typical cases with pleural recurrence and needle tract implantation have been identified since 1965 when PTNB was firstly reported to be used^4^,^5^,^21^,^22^,^23^. A cohort study, however, was lack until 2005 Matsuguma, *et al*. reported that PTNB increased pleural recurrence in patients with stage I lung cancer^7^. Following Matsuguma, *et al*., several studies focused on this topic were reported^8^,^9^,^10^,^11^,^24^. Another study by Wisnivesky *et al*. reported that the overall survival of patients after PTNB was similar with patients diagnosed with other strategies, which is consistent with our results. The results of our analysis demonstrate that the total recurrence and pleural recurrence are not significantly different between PTNB and non-PTNB group in patients with early stage lung cancer. To the best of our knowledge, this is the first meta-analysis focusing on this topic.

It is noteworthy that, despite the pooled analysis showed no difference on total recurrence and pleural recurrence, most included studies reported that patients in non-PTNB group had larger tumors and higher proportion of central tumor location than patients in PTNB group, which may cover and obscure the negative effect of PTNB on prognosis. It is possible that much earlier diseases with smaller tumor sizes might be included in the PTNB group which would result in better outcomes and false negative results. In addition, some studies included higher proportion of peripheral tumor location in PTNB group which might account for the higher incidence of pleural recurrence. Therefore, we restricted participants into stage I patients and performed the subgroup analysis according to tumor location. The result showed that PTNB would increase the risk of pleural recurrence in patients with sub-pleural lesions. But this risk was not found if the tumor was not located near the pleural. In PTNB groups, the pleural recurrence rate of patients with sub-pleural lesions (from 15% to 25%) was significantly higher than that of patients (from 0% to 12%) without sub-pleural lesions (Fig. 3), while in non-PTNB groups, the pleural recurrence rate of patients with sub-pleural lesions (from 2% to 4%) was similar with that of patients (from 2% to 9%) without sub-pleural lesions. No study included in our analysis reported available data to perform subgroup analyses according to tumor sizes, pathological subtypes, and other confounding factors.

There are some other limitations of this study. Our results are based on retrospective studies with small sample size, in most of which some important confounders such as tumor size, type of the surgery, adjuvant therapy, other diagnostic modalities, pathological subtype or baseline characteristics of patients could not be well adjusted. The evidence is low-level and a few studies were available to analysis, which is a major limitation. Also the median follow-up period of included studies were different and may lead to confounding factors for the limited number of event such as pleural recurrence or other recurrence. Additionally, different puncture methods could also affect the results of outcomes. In some studies, patients received needle puncture more than twice per procedure which might have increased the rate of pleural dissemination or recurrence compared with patients received only once. The present study included both fine needle and core needle biopsy methods, and the type of needle they used might influence the incidence of pleural recurrence. Actually, some significant heterogeneity was detected and most of it was unexplainable.

In conclusion, based on current evidences, PTNB is not associated with increased total recurrence and pleural recurrence in early lung cancer patients. But PTNB will increase the risk of pleural recurrence and should not be used in early patients with sub-pleural lesions. However, PTNB, as a minimally invasive procedure, is recommendable to use in other patients without sub-pleural lesions to whom pathological diagnosis is necessary, especially in patients with relevant comorbidities. Large scale, prospective, and multicenter studies are needed.

## Additional Information

**How to cite this article**: Wang, T. *et al*. Risk of Pleural Recurrence in Early Stage Lung Cancer Patients after Percutaneous Transthoracic Needle Biopsy: A Meta-analysis. *Sci. Rep.* **7**, 42762; doi: 10.1038/srep42762 (2017).

**Publisher's note:** Springer Nature remains neutral with regard to jurisdictional claims in published maps and institutional affiliations.

## Figures and Tables

**Table 1:** Characteristics of included studies.

Study	Study type	Location	Sample size		Period	Median follow-up (months)	Stage	Tumor size (cm)		Type of biopsy	Therapy	Quality scores
			PTNB	Non-PTNB				PTN B	Non-PTN B			
Matsuguma H 2005	Cohort study	Japan	66	224	1986–2000	80	I	2.38	2.90/3.95	CCNB 18 G	Surgery	7
Inoue M 2011	Cohort study	Japan	131	316	1992–2008	60.4	I	2.5	2.7	CCNB	Surgery	8
Asakura K 2012	Cohort study	Japan	124	197	2002–2009	45/42	I	1.9	2.5	CCNB 18 G	Surgery	8
Flechsig P 2015	Cohort study	Germany	26	9	2003–2010	17	I	NR	NR	CCNB 15 G	Surgery, chemotherapy, radiotherapy	6
Kashiwabara K 2016	Cohort study	Japan	63	86	2009–2014	43.2	I	2.1	2.7	FNB 21 G	Surgery	8

*PTNB: percutaneous transthoracic needle biopsies; CCNB: CT-guide transthoracic core needle biopsy, CNB: fine needle biopsy.
